# Folic Acid-Decorated Nanocrystals as Highly Loaded Trojan Horses to Target Cancer Cells

**DOI:** 10.1021/acs.molpharmaceut.3c01186

**Published:** 2024-04-27

**Authors:** Marta G. Fuster, Jiawen Wang, Octavio Fandiño, Gloria Víllora, Alejandro J. Paredes

**Affiliations:** † School of Pharmacy, Queen’s University Belfast, Medical Biology Centre, 97 Lisburn Road, Belfast BT9 7BL, U.K.; ‡ Department of Chemical Engineering, Faculty of Chemistry, University of Murcia (UMU), Campus de Espinardo, Murcia 30100, Spain

**Keywords:** nanocrystals, surface decoration, EDC/NHS chemistry, nanosuspensions, media milling

## Abstract

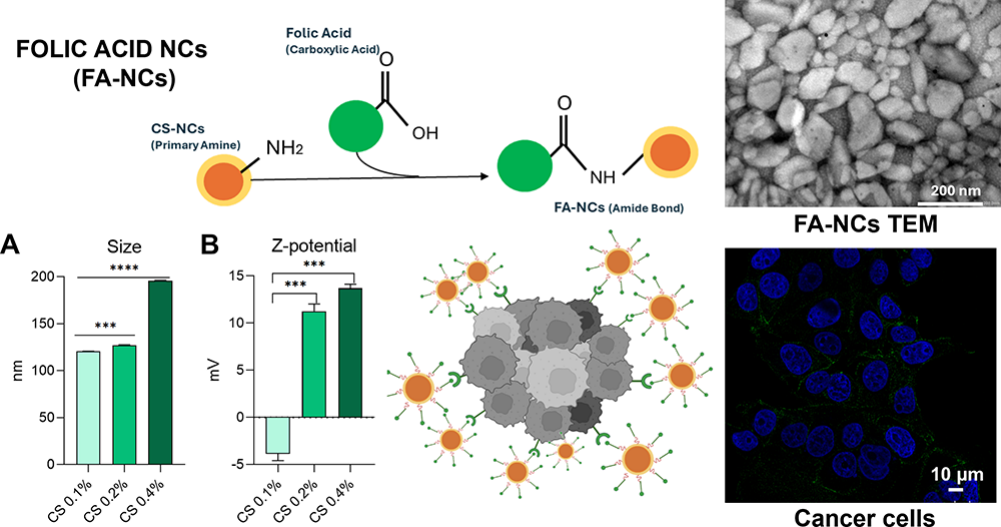

The nanocrystal (NC) technology has become one of the most commonly used strategies for the formulation of poorly soluble actives. Given their large specific surface, NCs are mainly used to enhance the oral absorption of poorly soluble actives. Differently from conventional nanoparticles, which require the use of carrier materials and have limited drug loadings, NCs’ drug loading approaches 100% since they are formed of the pure drug and surrounded by a thin layer of a stabilizer. In this work, we report the covalent decoration of curcumin NCs with folic acid (FA) using EDC/NHS chemistry and explore the novel systems as highly loaded “Trojan horses” to target cancer cells. The decorated NCs demonstrated a remarkable improvement in curcumin uptake, exhibiting enhanced growth inhibition in cancer cells (HeLa and MCF7) while sparing healthy cells (J774A.1). Cellular uptake studies revealed significantly heightened entry of FA-decorated NCs into cancer cells compared to unmodified NCs while also showing reduced uptake by macrophages, indicating a potential for prolonged circulation *in vivo*. These findings underline the potential of NC highly loaded nanovectors for drug delivery and, in particular, for cancer therapies, effectively targeting folate receptor-overexpressing cells while evading interception by macrophages, thus preserving their viability and offering a promising avenue for precise and effective treatments.

## 1 Introduction

Nanoparticles have emerged as revolutionary tools in drug delivery, offering a versatile platform for the encapsulation and transportation of therapeutic agents. Their small size, typically falling within the range of 1 to 100 nm, enables unique physicochemical and biological properties, such as an augmented reactive surface area and the ability to be readily taken up by cells, allowing for an efficient delivery of currently available bioactive compounds.^1^ Notable examples of such nanocarriers include liposomes,^2^ solid lipid nanoparticles,^3^ dendrimers,^4^ polymers,^5^ silicon or carbon materials, and magnetic nanoparticles,^6^ all of which have been extensively explored as drug delivery systems.

While nanoparticles present a multitude of advantages in biomedicine, they are not free of limitations. One notable challenge arises from their lack of specificity, potentially leading to unintended effects by not exclusively targeting the intended cells or tissues, reminiscent of issues encountered in conventional treatment methods.^7^ Additionally, both conventional drug delivery methods and nanoparticle-based approaches share inherent limitations such as constrained drug bioavailability, resulting in suboptimal drug concentrations at the intended target site. This, coupled with the potential for nontargeted drug delivery, can result in adverse side effects as healthy tissues are exposed to the therapeutic agent. Addressing these limitations is the key to optimizing therapeutic outcomes and improving the overall quality of patient care.

New techniques based on targeting have been developed for this purpose. Targeting involves directing drugs specifically to their intended sites of action. In cancer treatment, this principle has taken on paramount importance. The ability to selectively target malignant cells while sparing healthy tissues can lead to a paradigm shift in oncological therapies. While cell-specific targeting can be achieved through active or passive mechanisms, the method of loading the drug to the nanocarrier and the targeting strategy are crucial for effective therapy. Passive targeting leverages enhanced vascular permeability and retention in leaky tumor tissues, whereas covalent linking provides precise control over the amount of the therapeutic compound delivered.^8^ Active targeting employs recognition ligands like antibodies,^9^ low-molecular-weight ligands^10^ [e.g., folic acid (FA), peptides, and mannose], or physical stimuli (e.g., temperature, pH, and magnetism),^11,12^ through which drugs can be effectively guided to their target sites. This approach markedly enhances specificity, reducing the risk of nontargeted interactions and side effects. Another important limitation of conventional nanocarriers pertains to the constrained drug payload that they can carry, usually between 5 and 20%.^13^ The finite capacity to encapsulate therapeutic agents can restrict further development and clinical translation.

In the quest for optimized drug delivery, NCs have emerged as a promising innovation. These crystalline particles, operating on the nanoscale, predominantly consist of a pure drug and are surrounded by a thin layer of a stabilizer, thus reaching a drug loading that approaches 100%.^14^ Initially used to enhance the oral absorption of poorly soluble agents, NCs have been explored for the administration of hydrophobic drugs via multiple administration routes, with >20 NC-based products in the market.^15^ By reducing the drug particle size to the nanometer range, NCs hold the potential to enhance the drug dissolution rate, bioavailability, and adhesion to biological surfaces substantially due to their enlarged surface.^16^ Although NCs are designed to dissolve quickly in the large volume of fluids in the stomach, i.e., when administered orally, the literature indicates that they can circulate as integral particles in the body for variable periods of time, depending on the administration route.^17,18^ Therefore, decoration of the NCs’ surface with specific ligands results in an interesting target for exploration since active targeting of cells and tissues can be achieved before NC dissolution.

Curcumin (CUR), a poorly soluble natural polyphenol renowned for its anti-inflammatory, antioxidant, and anticancer properties,^19,20^ was used as a model drug in this paper. Crucially, formulating CUR as NCs (CUR-NCs) ameliorates its dissolution rate and allows for a more efficient drug delivery.^21,22^ In a major departure from traditional uses of NCs as a dissolution enhancing strategy, this paper proposes the functionalization of CUR-NCs with FA to promote folate receptor-mediated targeting of cancer cells.^23−25^

**Figure 1:**
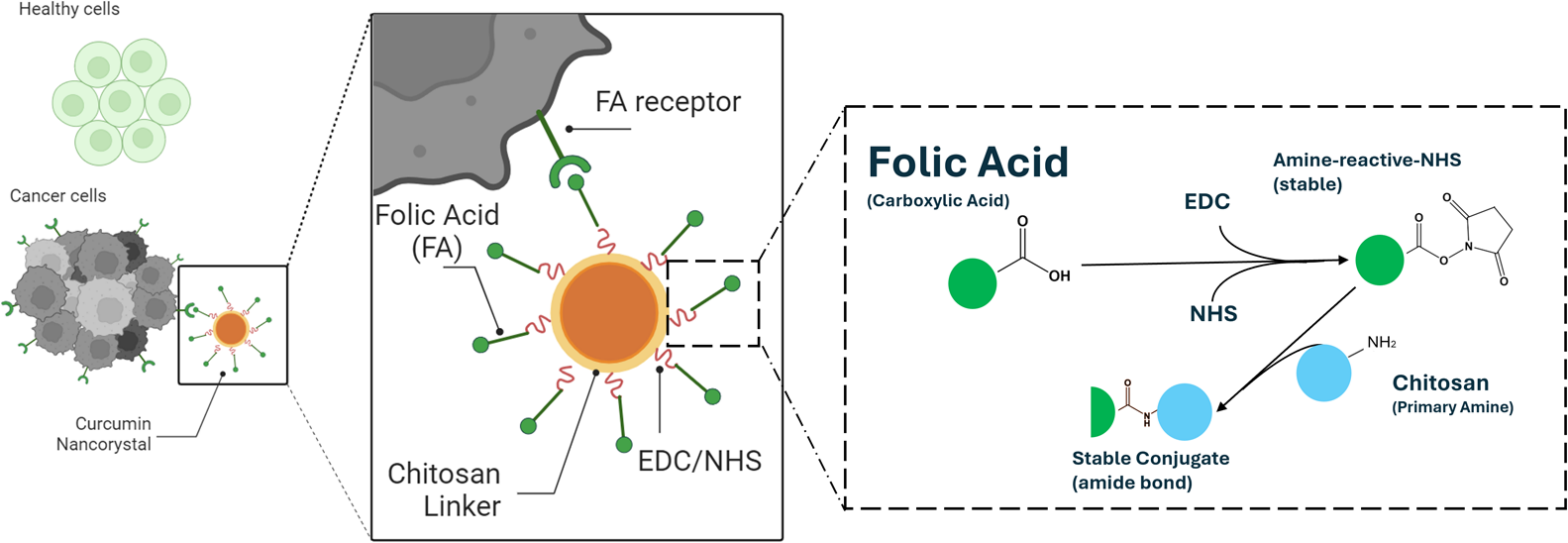
Mechanism of action of folic acid-functionalized NCs using an innovative procedure in this field on cancer cells. Selective drug accumulation in cancer cells and evasion of macrophage internalization would prevent damage to healthy cells and enhance cytotoxicity on cancer cells.

This paper describes the formulation of CUR-NCs by media milling and, for the first time, reports the decoration of the NCs’ surface covalently using EDC/NHS chemistry. The proposed system and its application are illustrated in Figure 1.

## 2 Materials and Methodologies

### 2.1 Materials

CUR was purchased from Cayman Chemical Industries (United Kingdom). For the milling process, yttria-stabilized zirconia beads (type YTZP) of 0.1–0.2 mm diameter were purchased from Chemco International (Guangfu, China). Chitosan middle-viscous was from Fluka Biochemika. Magnetic stirring bars (25 × 8 mm), d-α-tocopheryl polyethylene glycol succinate (TPGS), gelatin, acetonitrile (ACN) (>99.9%), methanol (MeOH) (>99.9%), *N*-hydroxysuccinimide (NHS), dimethyl sulfoxide (DMSO), and 1-[3-(dimethylamino)propyl]-3-ethylcarbodiimide methiodide (EDC) were purchased from Sigma-Aldrich (Dorset, UK). Phosphate-buffered saline (PBS) pH 7.4 tablets were purchased from Oxoid Limited (Hampshire, UK). High-performance liquid chromatography (HPLC) water was obtained from a water purification system, Elga Purelab DV 25, Veolia Water Systems (Ireland). A 0.2 μm MF-Millipore membrane was from EMD Millipore (Darmstadt, Germany). Parafilm M was purchased from Bemis Company, Inc. (Neenah, USA). All other chemicals used were of analytical grade.

### 2.2 Preparation of CUR-NCs

A media milling technique utilizing an ultrasmall-system assembly that was previously reported was applied to prepare NCs, with minor changes.^21,22,26−29^ Chitosan (CS) in combination with TPGS was used to stabilize the NCs (CS-NCs), which enabled to have −NH_2_ groups available on the NCs’ surface for subsequent drafting with FA, as detailed in the following section. To obtain the NCs, 100 mg of CUR, 5 mL of 0.5% w/v TPGS, and 3 mL of 0.1, 0.2, or 0.4% w/v CS were added into a 12 mL glass vial together with 3 mL of yttria-stabilized zirconia beads and two magnetic stirring bars of 25 × 8 mm (IKAFLON, IKA, Staufen, Germany). The system was hermetically sealed and wrapped with aluminum foil to protect the drug from light. The vials were then attached to an IKA RCT basic magnetic stirrer (Staufen, Germany) using adhesive tape and agitated at 1200 rpm for 24 h. Afterward, the NC suspensions were separated from the beads and magnets using a 200 mesh sieve (74 μm pore size). Following this, 1 mL of the resultant nanosuspensions was centrifuged for 15 min at 14,462*g* (Sigma microtube centrifuge, SciQuip Ltd., Shropshire, UK), and the pellet was resuspended in 0.5 mL of water. This allowed concentration of the sample and removal of any polymer and surfactant excess while keeping only the polymer attached to the NCs’ surface. The suspensions were stored in dark conditions for subsequent functionalization as detailed in the following section. A control formulation consisting of NCs stabilized only with 0.5% w/v TPGS (CUR-NCs) was prepared using the same experimental approach described above.

### 2.3 Preparation of Folic Acid-Functionalized NCs

**Figure 2:**
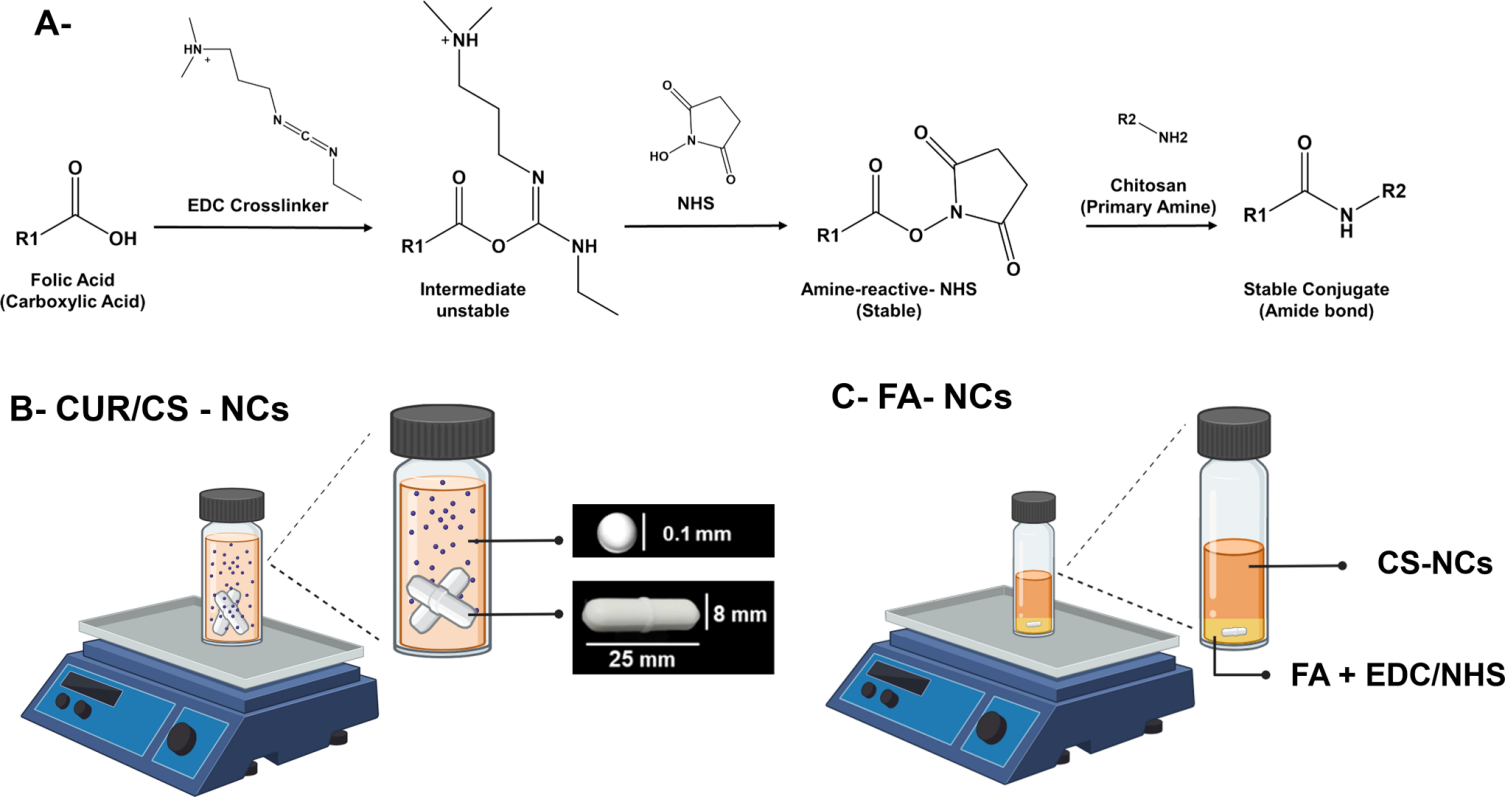
Illustrative description of the experimental setup used for the preparation of NCs. (A)Chemical reaction describing the formation of an amide bond between amino groups of chitosan and folic acid using EDC/NHS chemistry. (B) CUR/CS-NCs using media milling. (C) Functionalization of NCs by a chemical approach using EDC and NHS reagents.

Functionalization of drug NCs with FA was carried out in two stages to obtain FA-NCs. The first consisted of the activation of the carboxyl groups of FA followed by covalent bonding of the NCs (via the −NH_2_ groups on their surface) to the FA. First, 15 mg of FA, 3.75 mg of NHS, and 2.25 mg of EDC were added to an 8 mL vial with 3 mL of Milli-Q water to facilitate the reaction.^11^ The mixture was magnetically stirred for 40 min. Subsequently, 3 mL of CS-NCs was added to the activated FA and stirred for 24 h to allow the chemical reaction described in Figure 2A. Finally, 1 mL of the obtained sample was centrifuged for 15 min at 14,462*g* (Sigma microtube centrifuge, SciQuip Ltd., Shropshire, UK) and resuspended in 0.5 mL of Milli-Q water. The system was hermetically sealed and wrapped with aluminum foil to protect the drug from light. An illustrative representation of the experimental setup is presented in Figure 2.

#### 2.3.1 Particle Size, Size Distribution, and Zeta Potential

The particle size, polydispersity index, and zeta potential of all samples were measured by a Nanobrook Omni dynamic light scattering (DLS) analyzer. All samples (20 μL) were diluted with distilled water (2.5 mL) in the measurement cuvettes and gently mixed by hand. The equilibration time was set at 3 min, and determinations were made at 25 °C. All the samples were measured in triplicates and recorded as mean values ± standard deviation (mean ± SD, *n* = 3).

#### 2.3.2 Attenuated Total Reflectance-Fourier Transform Infrared Spectroscopy (ATR-FTIR)

The chemical compositions of nanoformulations and raw materials were compared using an FTIR spectrometer (FT/IR-4100 series, Jasco, Essex, UK) in the wavelength range of 4000 to 500 cm^–1^ for ATR-FTIR spectra.

#### 2.3.3 Transmission Electron Microscopy (TEM) and Field-Emission Scanning Electron Microscopy (FESEM)

The morphological characterization of the nanoparticles was performed by FESEM using an FEI Scios field-emission scanning electron microscope (Thermo Scientific, Waltham, MA, USA) operated at 20 kV. Samples of 5 μg/mL were dispersed in water by sonication; a drop was placed on a pedestal and dried by infrared; and then, it was coated with platinum. The morphology of the NCs was also analyzed by using a transmission electron microscope (Jeol, Japan). Before measurement, a droplet of the NC suspension was carefully deposited onto a copper grid that had been previously coated with a carbon film. To facilitate scanning during analysis, a negative staining agent, specifically a 1% (w/v) uranyl acetate solution, was applied to the copper grid.^30^ The grid underwent a drying process at room temperature under atmospheric pressure for several hours before the commencement of the observation.

### 2.4 Nanocrystal Drug Content

One hundred μL of the NC sample was diluted in 900 μL of ACN and mixed thoroughly using a vortex for at least 1 min. Subsequently, centrifugation was applied using a Sigma microtube centrifuge (SciQuip Ltd., Shropshire, UK) at 14,462*g* for 15 min. Following centrifugation, 20 μL of the supernatant was transferred and added to 1980 μL of ACN. After centrifugation, all samples were filtered through a 0.2 μm filter cartridge before injection into the HPLC system. The results were analyzed using the method described in Section 2.6.

### 2.5 Folic Acid Conjugation Efficiency

The concentration of FA in decorated NCs was measured by using UV–vis spectrophotometry (Rigol Ultra-3600, Rigol, Suzhou, China) following previously reported protocols.^31,32^ A calibration curve of FA in DMSO was prepared at a concentration range of 2.5–30 μg/mL, and determinations were made at a λ_max_ of 302 nm. Further details of the analytical method are presented in the Supporting Information. After the functionalization and purification steps, decorated NCs were centrifuged for 30 min at 14,000*g* in plastic tubes, and resultant pellets were dissolved in DMSO to determine the concentration of FA. The conjugation efficiency was estimated using eq 1:^33^
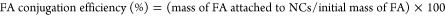
1

### 2.6 High-Performance Liquid Chromatography (HPLC) Analysis

The detection and quantification of CUR were performed by using reversed-phase HPLC-UV following the protocol reported by Zhang et. al.^34^ A ZORBAX Eclipse XDB-C18 column (50 × 4.6 mm internal diameter; 1.8 μm particle size) was selected as the stationary phase. The mobile phase was composed of 80:20% (v/v) ACN and water (0.1% phosphoric acid). The maxima absorption (λ_max_) was fixed at 425 nm. The injection volume was set as 20 μL, and the flow rate was 0.5 mL/min. The linearity of the method was explored between 0.1 and 50 μg/mL (*r*^2^ = 0.9999). The limits of detection and quantification were 0.27 and 0.81 μg/mL, respectively.

### 2.7 Proton Nuclear Magnetic Resonance (^1^H NMR)

^1^H NMR spectra were obtained using a Bruker UltraShield 400 Plus spectrometer. Samples were prepared by dissolving 5 mg of each NC (CUR-NCs, CS-NCs, and FA-NCs) in 2 mL of deuterated dimethyl sulfoxide in a vial. Eigh hundred μL of each solution was placed in a 5 mm precision NMR tube. Measurements were performed at 30° pulses and a relaxation delay time of 1 s using 16 scans. The spectral width selected was 8013 Hz, and the temperature of the collection was 298 K. Deuterated dimethyl sulfoxide was used as an internal reference for the acquisition of the spectra, and the time resulting for each measure was approximately 4.09 s.

### 2.8 *In Vitro* Characterization in Cell Cultures

#### 2.8.1 Cell Cultures

Human cervical cancer cells (HeLa), human breast carcinoma cells (MCF7), and the mouse macrophage cell line (J774A.1) were purchased from the American Type Culture Collection (ATCC, Manassas, VA, USA), validated, and stored according to the supplier’s guidelines. Cells were kept in Dulbecco’s modified Eagle medium (without phenol red) enhanced with 10% (v/v) thermally deactivated fetal bovine serum, 1 mM glutamax, 1 mM pyruvate, and 1% penicillin-streptomycin. Cancer and healthy cells were cultured in a humidified environment with 5% and 7% CO_2_, respectively. At 37 °C, they were cultivated in 75 cm^2^ culture flasks. After some passages, the cells were seeded for the tests, and when they achieved confluence, they were removed using a solution of 0.25% trypsin-EDTA.

#### 2.8.2 Cytotoxicity Assay

The cytotoxic effects of CUR-NCs, CS-NCs, and FA-NCs on HeLa, MCF7, and J774A.1 cells were tested using trypan blue. Cells were seeded in 24-well plates at a concentration of 8 × 10^4^ cells/well. Twenty-four hours later, the cells were fed with a fresh medium that contained different concentrations of NCs at 100, 50, and 25 μg/mL. The experiment involved the use of a control growth medium that did not contain NCs. After 24 h, cells were resuspended in a complete growth medium. Cells were also stained with trypan blue (100 μL of the cell suspension and 100 μL of 0.4% trypan blue), incubated for 2 min at room temperature, and counted using a TC20 automated cell counter (Bio-Rad, Inc., Hercules, California). Each sample was tested in at least three independent sets.

#### 2.8.3 Inverted Fluorescence Microscopy

To evaluate the cytotoxic effects of FA-NCs visually, tests were carried out at different exposure times. HeLa cells were seeded in 6-well plates at a ratio of 73,000 cells/mL culture medium and incubated at 37 °C with 5% CO_2_ for 24 h. The medium was then removed, and a dispersion of the FA-NCs was added at a concentration of 100 μg/mL. The cells were exposed to the NCs for 1–6 h, and then, photographs were taken at a 10× magnification with an inverted fluorescence microscope.

#### 2.8.4 Nanocrystal Cellular Uptake

The cellular uptake of HeLa, MCF7, and J774A.1 was determined for different NCs. For these assessments, 1.7 × 10^5^ cells/well were seeded into a 12-well plate and incubated for 24 h. The culture medium was replaced by a fresh medium with 100, 50, and 25 μg/mL NCs. After three washes with PBS, the cells were digested with trypsin to obtain cell suspensions. Cell-associated fluorescence was quantified by a Becton–Dickinson FACSCalibur flow cytometer (New Jersey, United States).

#### 2.8.5 Cell Cycle Arrest Assay

Studies of the effect of CUR-NCs, CS-NCs, and FA-NCs on cell cycle arrest were performed on HeLa, MCF7, and J774A.1 cell lines. A total of 1.7 × 10^5^ cells/well were seeded in 12-well plates and allowed to fix to the plates for 24 h at 37 °C in a 5% CO_2_ and 95% humidity atmosphere. Then, 100, 50, and 25 μg/mL were incubated for 24 h. Untreated cells were used as the control group. Cells were collected and centrifuged at 250*g* for 10 min. After incubation, we collected and centrifuged the cells at 250*g* for 10 min, washed them with PBS, and suspended them in 200 μL of PBS for further analysis. Subsequently, a PBS (30%) and ethanol (70%) mixed solution was added to the cells, and the cells were kept on ice for 30 min. The ethanol was then eliminated via centrifugation, and the cells were suspended in 400 μL of PBS, to which 50 μL of RNase solution and 50 μL of propidium iodide (PI) were added at final concentrations of 0.1 and 40 mg/mL, respectively. Following this, the cells were incubated in the dark for a period of 30 min. The PI fluorescence was measured for each cell in a Becton–Dickinson FACSCalibur flow cytometer. In each case, 20,000 events were acquired.

#### 2.8.6 Confocal Microscopy

To confirm cellular uptake of nanoparticles, confocal laser scanning microscopy imaging was performed on HeLa and J774A.1 cell lines. Cells (3 × 10^4^) were seeded on plates for 24 h. After 4 h, CUR-NC and FA-NC cells were washed with PBS 1×. In addition, MCF7 cells were seeded and washed with PBS 1× and fixed for 10 min in 4% paraformaldehyde. After several washes with PBS, the nuclei of the cells were stained with DAPI (4′,6-diamidino-2-fenilindol) (Sigma-Aldrich, St. Louis, MO, USA). Confocal images were obtained with a Leica STELLARIS 8 inverted confocal laser scanning microscope, a white laser, and a FRET-FLIM module.

### 2.9 Statistical Analysis

The data were statistically analyzed by GraphPad Prism version 9 (GraphPad Software, San Diego, California, USA). An unpaired *t*-test was applied when comparing two cohorts, whereas one-way ANOVA was applied to compare more than two cohorts. The results were expressed as means ± SD, and in all cases, a *p* value <0.05 denoted significance.

## 3 Results and Discussion

### 3.1 Production and Characterization of CS-NCs

**Figure 3:**
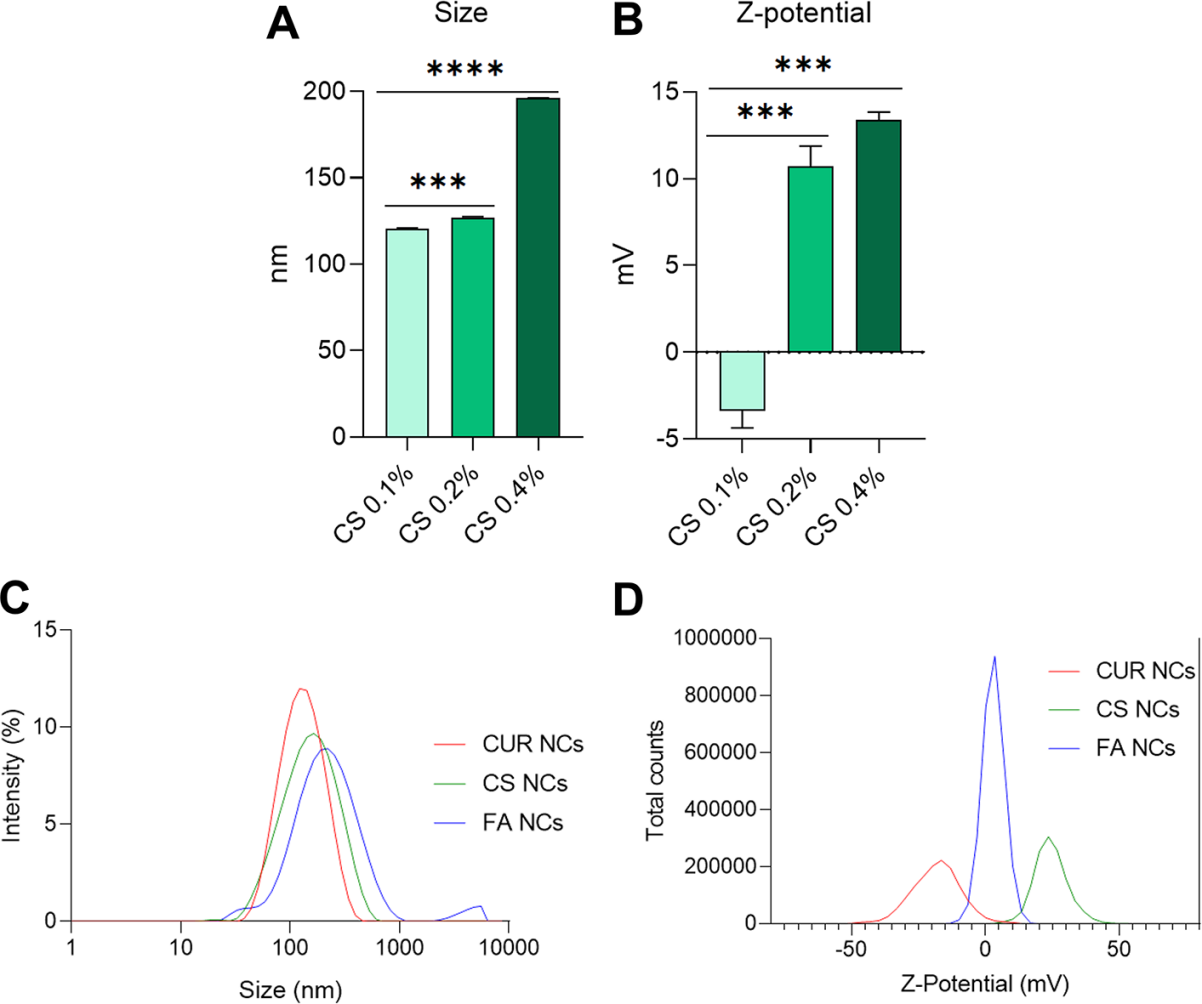
Size (A) and Z-potential (B) results of surface modification of CUR-NCs with different concentrations of CS. Size distribution based on the intensity (C) and Z-potential distribution of CUR-NCs (red), CS-NCs (green), and FA-NCs (blue). Infrared spectra. Results are presented as means ± SD (*n* = 3). *** indicates *p* < 0.001 and **** indicates *p* < 0.0001, compared to each other.

CUR-NCs, initially synthesized at a concentration of CUR of 13.63 mg/mL, underwent modification by introducing various concentrations of chitosan (0.1–0.4% w/v). This adjustment aimed to incorporate free amine groups on the nanocrystal surface, enhancing their potential for drug delivery applications. Upon closer examination, it was observed that CS-NCs presented optimal properties, in terms of both size (129.3 ± 0.81 nm) and Z-potential (25.2 ± 0.75 mV), when the CS concentration reached 0.2% w/v. Beyond this concentration, the size of the NCs increased proportionally (Figure 3A). Additionally, Z-potential values underwent a significant shift from negative to positive as the chitosan concentration increased. This shift in Z-potential indicated the successful attachment of chitosan to the NCs, facilitated by the negatively charged nature of CUR, which resulted in electrostatic interactions (Figure 3B). This observation is consistent with the presence of numerous amino groups along the backbone of chitosan,^35^ leading to a positively charged surface and facilitating subsequent derivatization. Furthermore, the drug content within the CS-NCs was determined to be 11.24 mg/mL. This finding underscores the successful encapsulation and modification of CUR nanoparticles by using varying concentrations of chitosan, enhancing their potential for drug delivery systems. Importantly, the preparation of drug NCs using magnetically powered media milling stirring has been reported previously in the literature. Romero et al.^36^ described the use of 2 mL vials to produce NCs, whereas work from our group reported the manufacture of drug NCs of various drugs including CUR using 10 mL vials.^21,22,26−29^ In this process, the movement of the magnetic bars and milling media leads to the impact, shear and compression forces, and particle breakage.^37^ Since the milling occurs in a solution of the surfactant, the newly created surfaces are quickly stabilized by the polymer/surfactant molecules that place their hydrophobic moieties on the NCs’ surface by electrostatic forces.^38^ The fact that the NCs could stand the centrifugation process and redisperse with similar nanoparticle properties (size and charge) is an indication that the electrostatic interaction between chitosan and the NCs’ surface is strong. A similar case was reported by Abbate et al., where NCs of rilpivirine and cabotegravir were coated with chitosan.^28^ This is crucial to this work, as the chitosan molecules stabilized on the NCs’ surface allowed further functionalization with FA as described in the following section.

### 3.2 Functionalization Procedures

To functionalize the NCs, we employed an approach that stands out in the field of chemical modification of conventional nanoparticles. This technique involved the use of EDC/NHS, a method renowned for its ability to covalently link a primary amine with a carboxylic group, resulting in the formation of an amide group. Notably, after functionalization, we examined the conjugation of FA. FA-NCs displayed a size of 177.8 ± 1.17 nm, slightly higher than that of CS-NCs, which indicates the successful NC drafting with FA. The reduction of the Z-potential to 2.7 ± 0.22 mV (Figure 3C) also supports the attachment of FA and a reduction of free −NH_2_ groups on the NCs’ surface. Crucially, a final particle size lower than 200 nm and a surface charge close to neutrality are suitable for their purpose of cellular internalization.^39^ Furthermore, these data align with our intended purpose for the NCs, which were originally (CUR-NCs) sized at 108.9 ± 1.04 nm and displayed a Z-potential of −17.7 ± 0.72 mV (Figure 3D). The conjugation efficiency of FA, as determined by UV–vis spectrophotometry, was 29.3 ± 6.1%, indicating significant conjugation of FA with NCs due to the presence of amino groups in chitosan and activation of carboxylic groups in FA, allowing conjugation by EDC/NHS chemistry. Additionally, the drug content within these modified NCs was quantified at 5.37 mg/mL, highlighting their potential for pharmaceutical applications.

### 3.3 Physicochemical Characterization

#### 3.3.1 Fourier Transform Infrared Spectroscopy

**Figure 4:**
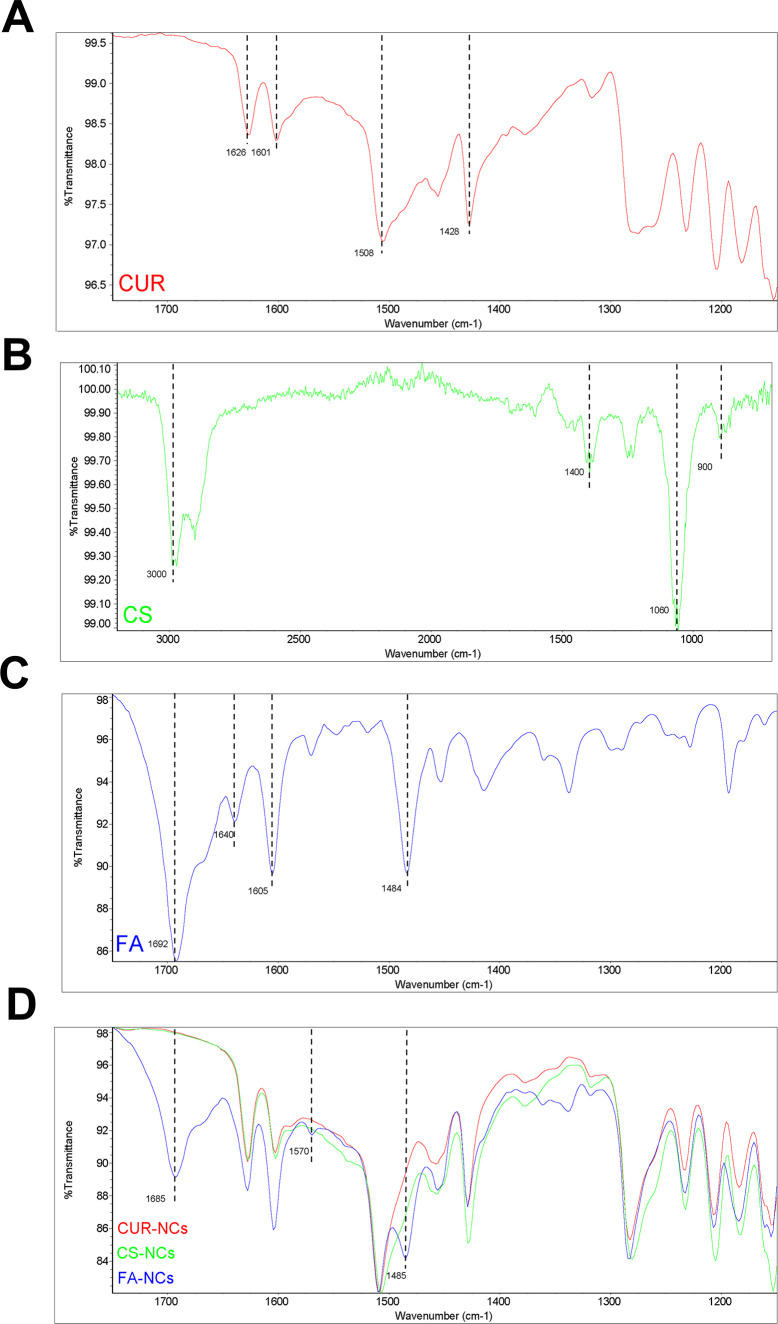
ATR-FTIR spectra of CUR (A), CS (B), and FA (C) and comparison spectra of CUR-NCs, CS-NCs, and FA-NCs (D).

The CUR-NCs, CS-NCs, and FA-NCs, together with CUR, CS, and FA, were analyzed by FTIR spectroscopy. Unprocessed CUR showed four characteristic peaks as can be seen in Figure 4A, with the most significant peak at 1626 cm^–1^ due to the stretching of C=O. Peaks at 1601 and 1508 cm^–1^ appeared due to aromatic C=C stretching. Phenolic C–O stretching appeared at 1428 cm^–1^, and the enolic C–O stretching peak appeared at 1274 cm^–1^.^40^ CUR-NCs showed five characteristic peaks of unprocessed CUR at 1628, 1603, 1508, and 1429 cm ^–1^. The FTIR spectrum of CS (Figure 4B) shows characteristic absorption of stretching where C–H was observed at 2950–3000 cm^–1^, C–H bending was recorded at 1350–1480 cm^–1^, N–H (amine) bending was at 900 cm^–1^, and C–N (alkyl) was at 1200–1025 cm^–1^.^41,42^ The presence of all major CUR peaks in CS-NCs ruled out any chemical interaction between CUR and chitosan (Figure 4D). This presumably implies that CS bonded to the NCs via a “layer-by-layer” charge difference approach. The FTIR spectrum of FA shows at Figure 4C that peaks at 1692 and 1605 cm^–1^ should be the peaks of the carboxyl group and aromatic C=C.^43^ After folate was grafted, the new peaks at 1685 (C=O stretching), 1485 (N–H in-plane bending), and 1570 cm^–1^ (N–H amide bond) appear in the spectrum of FA-NCs. The main reason for the appearance of these new peaks in FA-NCs with respect to CUR-NCs and CS-NCs (Figure 4D) is due to the formation of strong amide bonds produced by the chemical approach with EDC/NHS reagents involving attachment of the amine groups from CS to the free carboxyl groups from FA. Covalent drafting of other types of nanoparticles, i.e., mesoporous^44^ or polymer-coated,^45^ with FA for application in cancer has been reported before. Nonetheless, the fact that FA-NCs do not need a matrix or carrier materials presents a unique opportunity in terms of targeting with higher drug payloads. The data obtained in this section correlate with the changes in the particle size and zeta potential, all of them indicating successful modification of the NCs’ surface and attachment of FA covalently. However, in order to have further confirmation of this chemical reaction, ^1^H NMR analysis was carried out as presented in the following section.

#### 3.3.2 Proton Nuclear Magnetic Resonance (^1^H NMR)

**Figure 5:**
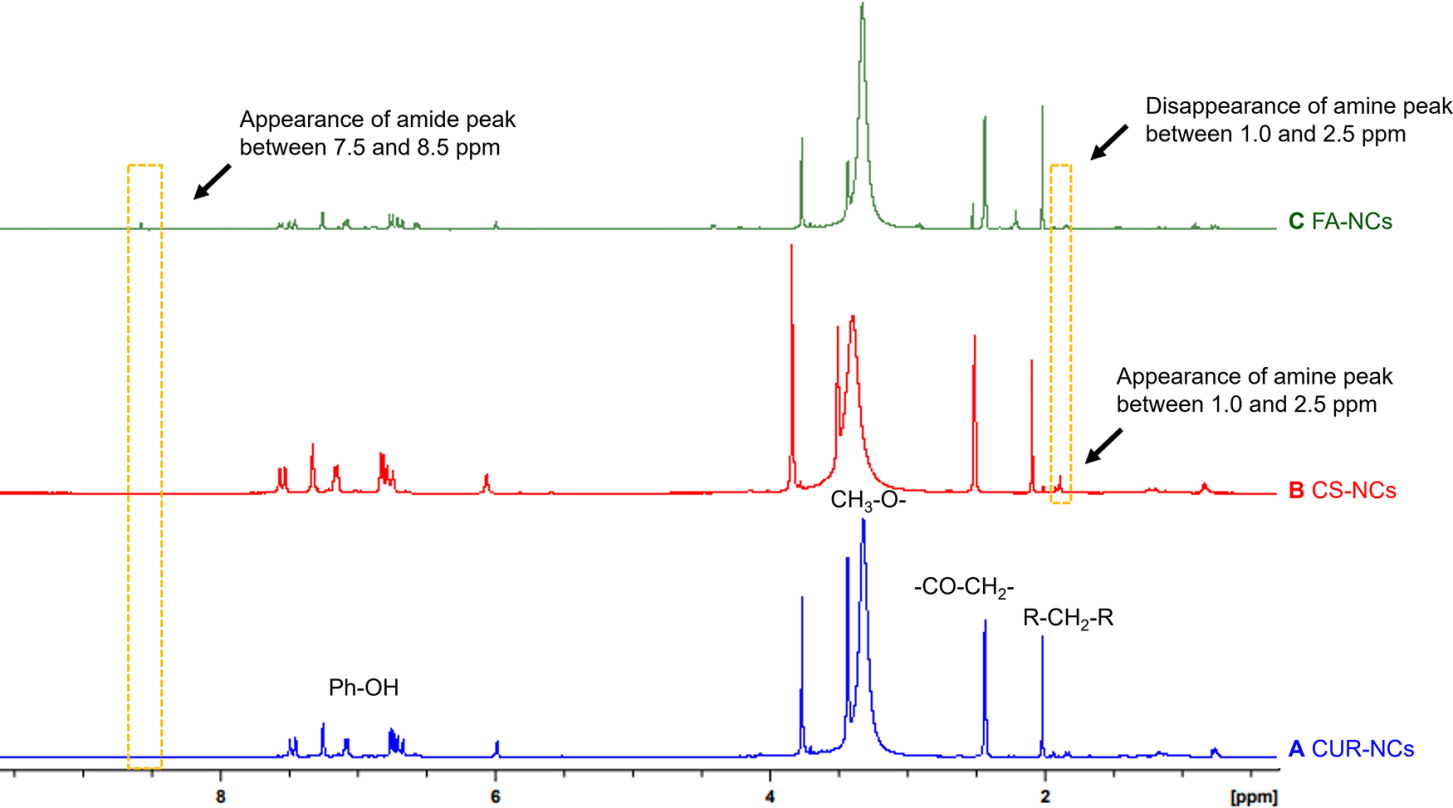
^1^H NMR spectra of CUR-NCs (A), CS-NCs (B), and FA-NCs (C) showing chemical shifts, which indicate the formation of amide groups between folic acid and amino groups from chitosan on the NCs’ surface. Additionally, characteristic functional groups of CUR are annotated in the CUR-NC spectrum, as it is the primary component of the mixture.^48^

^1^H NMR was used in the search for further evidence on the covalent attachment of FA to the NCs’ surface. As observed in the CS-NCs' (Figure 5B) ^1^H NMR trace, a distinctive band between 1.0 and 2.5 ppm emerges, indicative of the presence of an amine group, a characteristic moiety of CS^46^ located on the NCs’ surface prior to grafting with FA. In the FA-NCs (Figure 5C), the band corresponding to the amine group observed in vanishes, presumably indicating a reduction on the number of available amino groups. Crucially, this was accompanied by the appearance of a distinctive band at 7.5 to 8.5 ppm, characteristic of amide formation.^47^ This indicates that the proposed chemical reaction using EDC/NHS chemistry occurred and that FA was successfully attached to the amino groups of chitosan immobilized electrostatically on the NCs’ surface. These data, together with those of DLS and FTIR experiments, present a significant advancement to the field of NCs since covalent drafting of these nanoparticles has been rarely reported in the literature.

### 3.4 Nanocrystal Morphology

**Figure 6:**
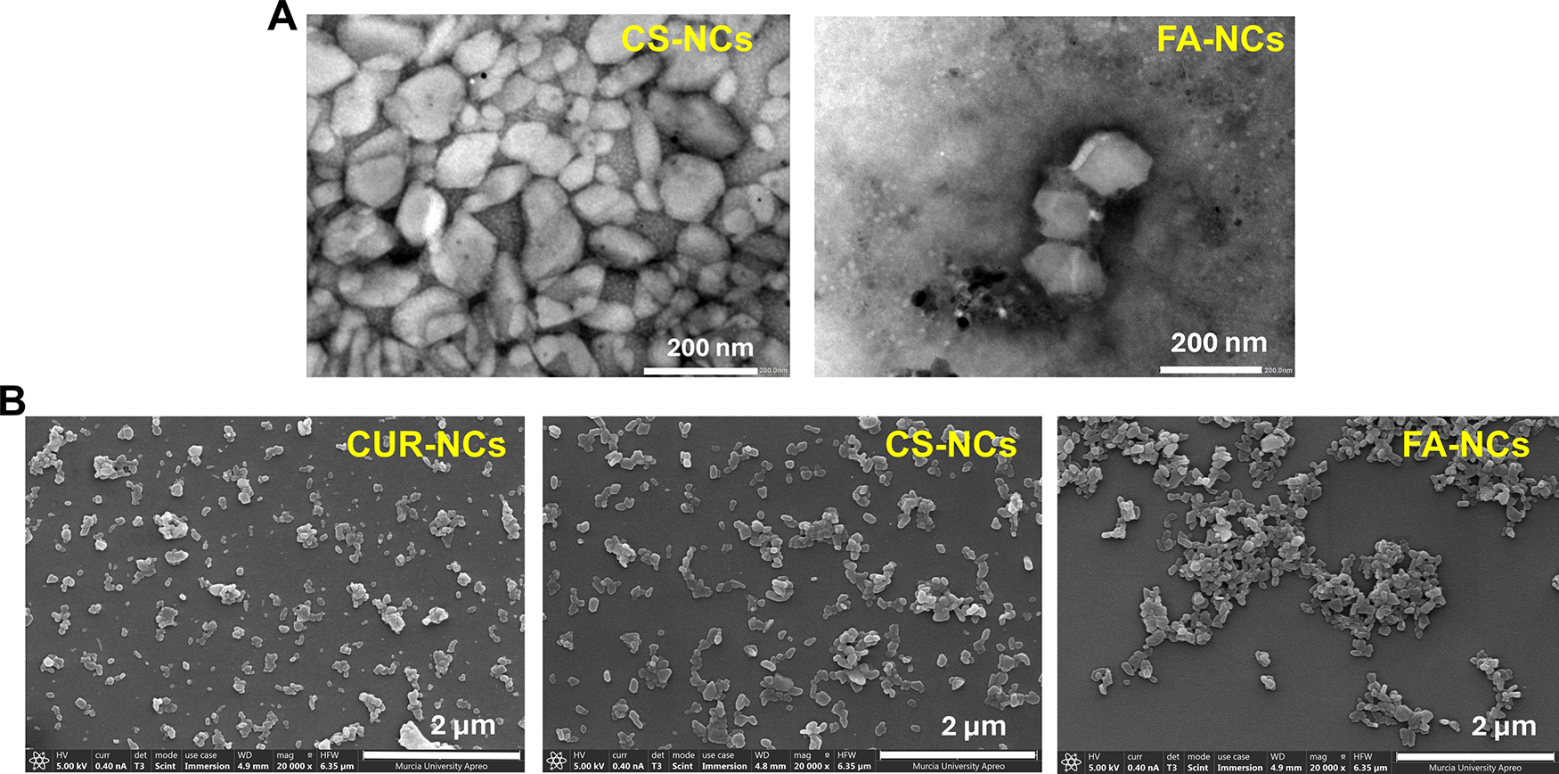
(A) Transmission electron microscopy (60,000×) and (B) field-emission scanning electron microscopy (20,000×) images of NCs.

The dimensions and morphology of the NCs were thoroughly examined through TEM and FESEM. Representative images of the system are depicted in Figure 6A,B. The NCs displayed a granular, nearly spherical morphology, with an approximate size of 100 nm. It is important to note that when the size observed through microscopy is compared with the *Z*-average measured using dynamic light scattering (DLS), several key factors should be considered. First, the DLS method derives size from diffusivity, presenting it as an intensity-weighted distribution, whereas microscopy provides size based on a number distribution. Second, in DLS, particles are dispersed in water, while in FESEM, the crystals are analyzed in their dry state. Consequently, in DLS, a swelling effect due to the interaction of particles with water must be considered.^49^ Considering these aspects, it became evident that the size measurements obtained through both techniques align consistently.

Importantly, previous reports where CUR was converted to NCs using similar media milling methods demonstrated that the process did not induce changes in the crystallinity of the drug, as per confirmed by powder X-ray diffractometry and differential scanning calorimetry.^21,22,50,51^

### 3.5 *In Vitro* Experiments in Cell Cultures

#### 3.5.1 Cytotoxicity Assay

**Figure 7:**
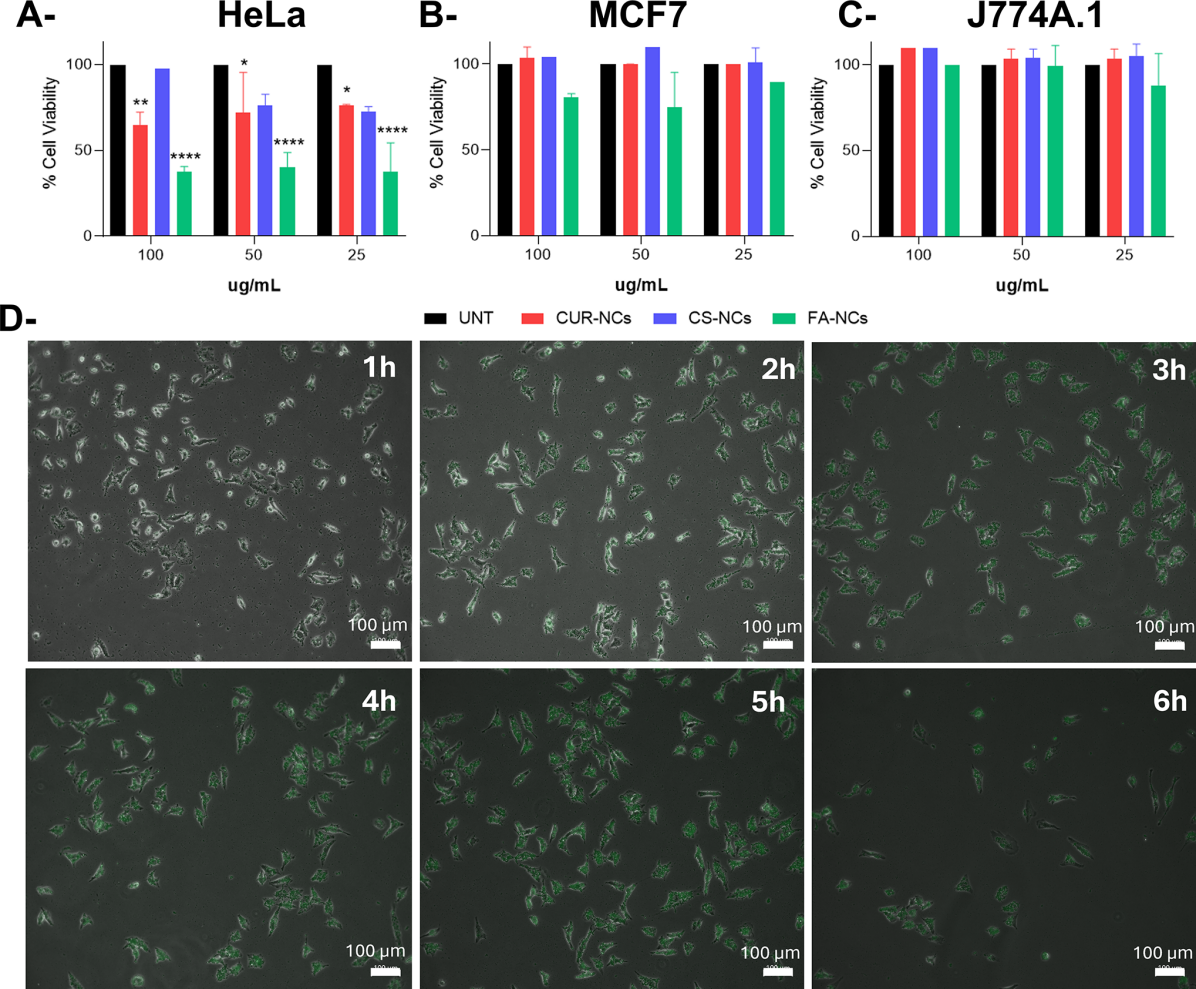
Cytotoxic effect on HeLa (A), MCF7 (B), and J774A.1 (C) exposed to 25, 50, and 100 μg/mL CUR-NCs, CS-NCs, and FA-NCs. Untreated (UNT) cells were used as a control. Data are expressed as the percentage of cell viability ± SD vs concentration. (D) Cytotoxic effect of FA-NCs on the HeLa cell line by inverted fluorescence microscopy analysis.

The cytotoxic effect of FA-decorated NCs was evaluated compared to those of CUR-NCs and CS-NCs on three different cell lines. Cervical carcinoma-derived HeLa cells were selected according to their origin and high-level expression of the folate receptor, a perfect target for the treatment study.^23^ In order to compare the results of these two cell lines with a control, the macrophage cell line J774A.1 was chosen due to its involvement in the first barrier of the immune system.^52^ The cytotoxicity of the different NCs was studied in three concentrations of CUR defined according to their drug load previously analyzed by HPLC, with promising findings. An increase in cytotoxicity was observed in the HeLa cell line (Figure 7A) when exposed to FA-NCs compared to nonfunctionalized NCs. Moreover, cell death did not increase in a concentration-dependent manner, meaning that the therapy could be used with the lower drug concentration to obtain high results. The cytotoxic effect was also assessed in the HeLa cell line at various time points. The results depicted in Figure 7D indicate that after 2 h of exposure, FA-NCs were detected inside the cells. By the 4 h mark, a decrease in the cell count due to the treatment was observed, and by 6 h, a substantial decrease in cell viability was evident demonstrating the effectiveness of treatment. For MCF7 cells (Figure 7B), a small decrease in cell viability (20%) was observed when exposed to FA-NCs. However, for CUR-NCs and CS-NCs, cells remained alive for all concentrations tested. This demonstrates that without the presence of FA, therapy in this cell type would not be successful. Furthermore, the results obtained were even more promising because of the viability of healthy J774A.1 cells (Figure 7C), which remained unshakable despite NC treatment even on functionalized ones. The overexpression of folate receptors in cancer cells has been well-reported in the literature,^53−55^ and there is a broad body of evidence demonstrating the feasibility of FA-mediated nanoparticles targeting cancer cells and tissues.^56−59^ Crucially, our results seem to point to the same direction since an increased internalization of FA-decorated NCs was observed in cancer cells, in comparison to nondecorated NCs, together with an overall lower NC internalization in macrophages. A similar trend was observed in MCF7 cells, which have been reported to express the folate receptor in a lower extent.^24,60,61^

#### 3.5.2 Cell Cycle Arrest

**Figure 8:**
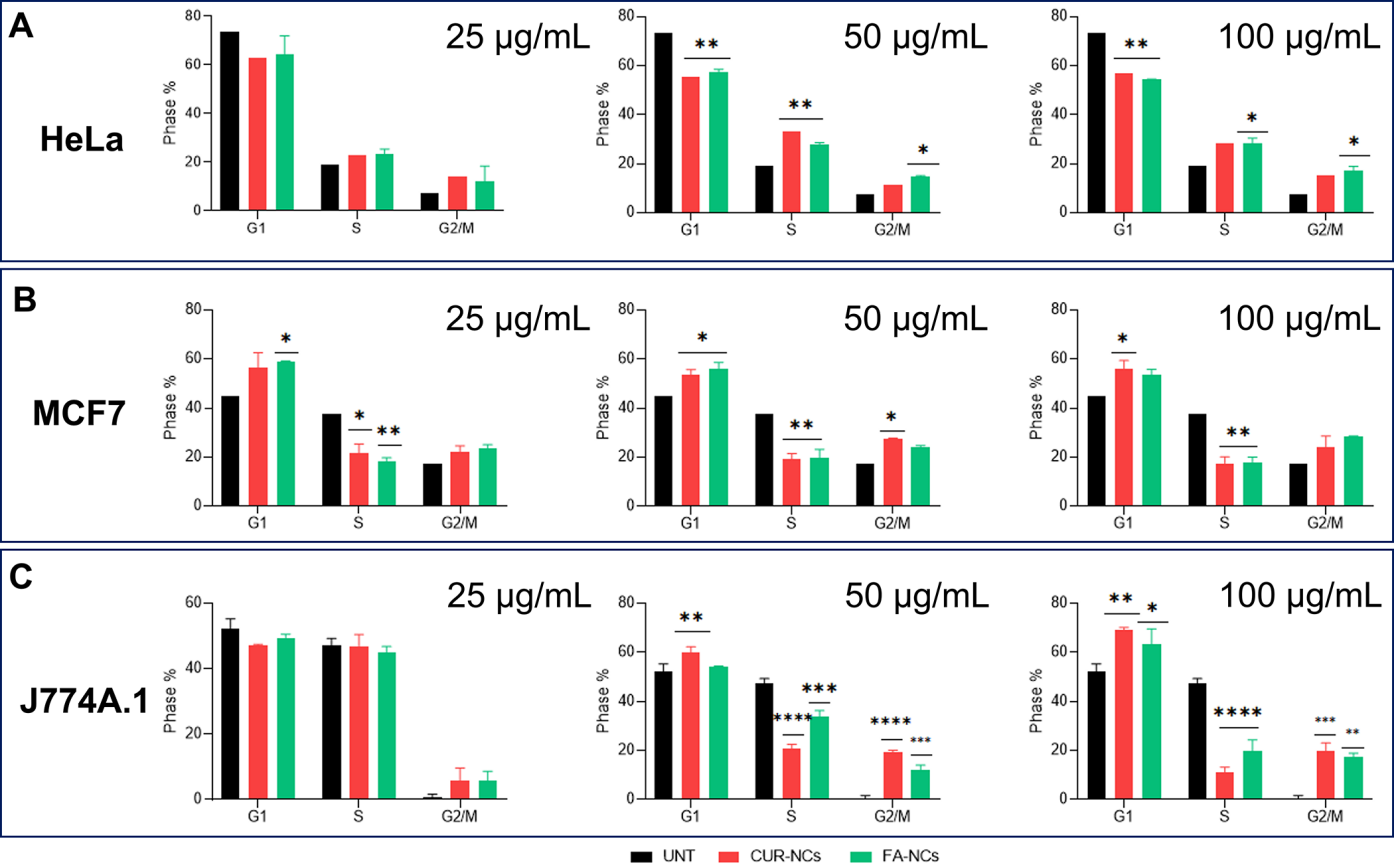
Cell cycle arrest on HeLa (A), MCF7 (B), and J774A.1 (C) exposed to 25, 50, and 100 μg/mL CUR-NCs, CS-NCs, and FA-NCs. Untreated (UNT) cells were used as a control. * indicates *p* < 0.05, ** indicates *p* < 0.01, *** indicates *p* < 0.001, and **** indicates *p* < 0.0001, compared to the control.

To study the inhibition of cell growth through cell cycle arrest, the effects of CUR-NCs, CS-NCs, and FA-NCs at 25, 50, and 100 μg/mL were examined on HeLa, MCF7, and J774A.1 cells for 4 h by flow cytometry and compered to the control. In HeLa cells (Figure 8A), it can be observed that as the concentration of the drug exposed to the cells increases, the percentage in the G1 phase decreases and increases in the later S phase. For instance, when the cells were exposed to 100 μg/mL CUR-NCs and FA-NCs, the G1 phase decreased significantly from 73.43 to 56.67 and 54.28%, respectively. The S phase increased significantly from 19.21 to 28.08% when exposed to CUR-NCs and 28.46% when exposed to FA-NCs. Finally, the G2/M phase also increased from 7.36 to 15.35 (CUR-NCs) and 17.25% (FA-NCs). These results suggest that HeLa cells treated with a higher concentration of highly loaded NCs were not able to overcome the G2 checkpoint, and therefore, the G2/M transition was affected. Cell cycle analysis for this cell line showed that NCs were able to arrest HeLa cells in the S phase, which could be associated with CUR-induced apoptosis in HeLa cells.^62^ The MCF7 cancer cell line showed equal cell cycle differences for all concentrations studied. As an example, as can be seen in Figure 8B, when cells were exposed to 100 μg/mL CUR-NCs and FA-NCs, the G1 phase increases from 44.74 to 55.89 and 53.56%, respectively. The S phase decreased from 37.76 (control) to 17.47 (CUR-NCs) and 17.94% (FA-NCs). Finally, the G2/M phase decreased from (control) 17.51 to 23.84% when exposed to CUR-NCs and 28.48% when exposed to FA-NCs. These results demonstrated that cell cycle arrest for MCF7 cells occurred in the G1 phase.^63^ According to Choudhuri et al.,^64^ despite the presence of elevated cyclin D1 levels on cancer cells, CUR treatment does not significantly disrupt its expression or interfere with its association with crucial proteins for carrying out the cell cycle such as Cdk4 or Cdk6. Consequently, CUR does not block cell cycle progression, suggesting that other pathways could be at play in prompting apoptosis. The results obtained for the cell cycle of normal J774A.1 macrophage cells are in agreement with the literature.^65^ Since the cell cycle of healthy cells (Figure 8C) is modified in order to regulate the disruptive parameters, these cycle modifications, as can be seen in the cytotoxicity discussed above, do not result in cell death. It is a normal process that such cells go through. These results supported by the literature demonstrate the substantial complexity of cancer and its treatment in this case with NCs. Summarizing, no significant differences were observed in the study of cell cycle arrest when comparing uncoated NCs (CUR alone) with coated NCs (containing FA), as demonstrated in the results above. Therefore, and as per the results above, cell cycle arrest for these cancerous cell lines was attributed to CUR, while FA appears to play no significant role in this experiment.

#### 3.5.3 Detection and Quantification of the NC Uptake

**Figure 9:**
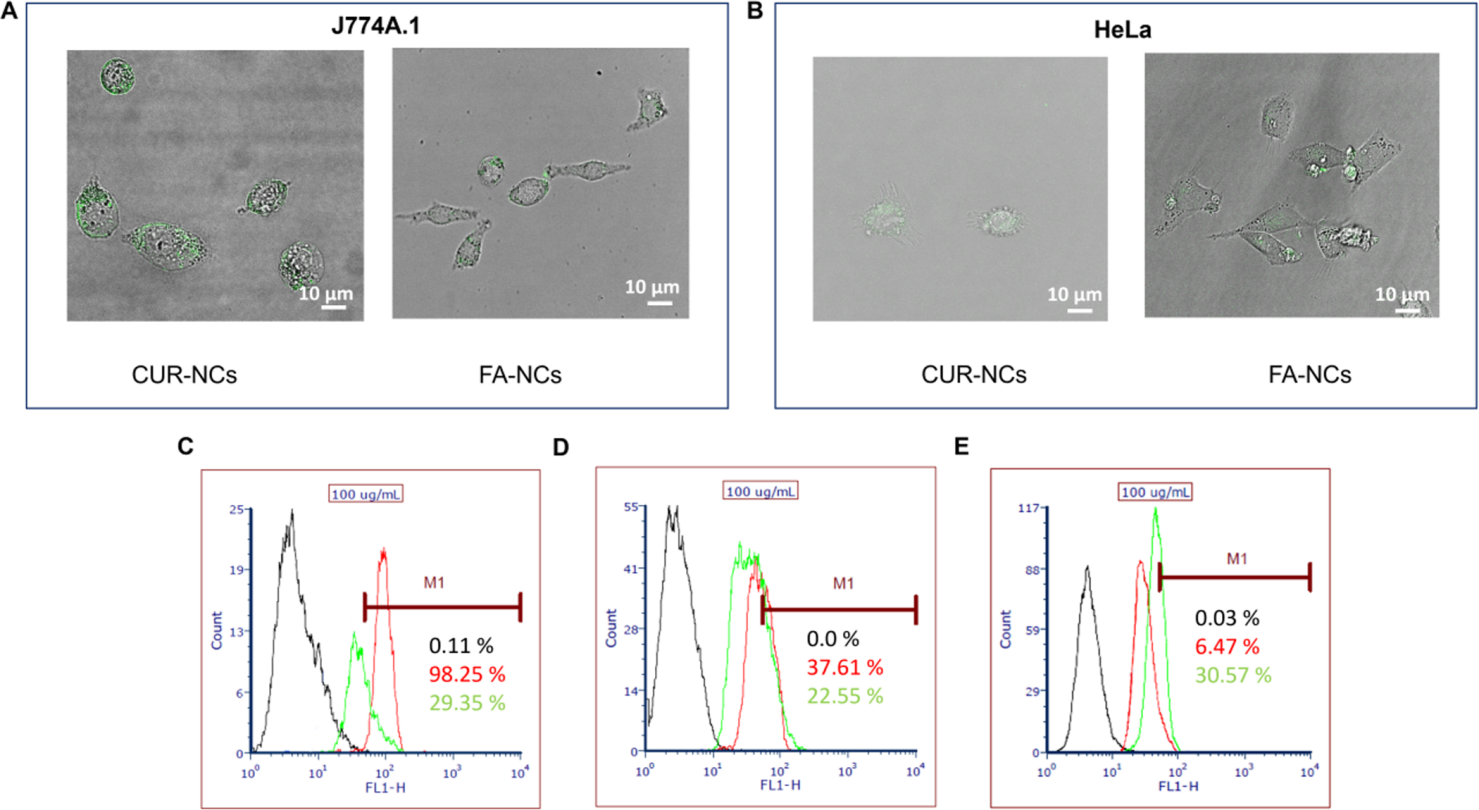
Detection by confocal microscopy of J774A.1 (A) and HeLa (B) cell lines of CUR-NCs and FA-NCs. Quantification by flow cytometry of J774A.1 (C), MCF7 (D), and HeLa (E) cellular uptake of CUR-NCs (red) and FA-NCs (green) compared to the control (black).

To assess the potential of functionalized NCs as targeted drug carriers, the cellular localization of CUR-NCs and FA-NCs was evaluated in HeLa and J774A.1 cell lines. These cell lines were chosen to represent cancer cells with high folate receptor expression (HeLa) and a healthy cell line (J774A.1). In both cases, the cellular uptake of NCs was observed by using confocal laser scanning microscopy. In the HeLa cell line (Figure 9B), an increase in NCs uptake was observed when they were functionalized, depicted in green due to the fluorescence of CUR. Importantly, it was very promising to observe that in the case of healthy cells (Figure 9A), there was a decrease in fluorescence when exposed to functionalized NCs compared to nonfunctionalized. This demonstrates that the developed FA-NCs would not be intercepted by macrophages, allowing them to progress until they reach cancer cells.^66^

Finally, the quantity of NCs entering the cells after 4 h of exposure was quantified by flow cytometry. In MCF7 cells (Figure 9D), a percentage difference in entry was observed, with 37.61% for CUR-NCs and 22.55% for FA-NCs. In HeLa cells (Figure 9E), there was a very significant increase in the entry of FA-NCs compared to CUR-NCs, from 6.47 to 30.57%, respectively. Lastly, a highly significant decrease in the entry percentage was observed in J774A.1 cells (Figure 9C) when exposed to functionalized NCs (29.35%) compared to nonfunctionalized ones (98.25%). These results align with the previously mentioned findings regarding cytotoxicity. There is greater cytotoxicity observed in HeLa cells when treated with FA-NCs compared to MCF7 cells. Furthermore, there is also an increased entry of NCs into these cells compared to MCF7 cells. This is attributed to the higher expression of folate receptors in HeLa cells compared to MCF7 cells.^67^ Therefore, as the treatment is based on FA binding, it suggests that the entry and subsequent drug action occur via the folate receptor pathway.

**Figure 10:**
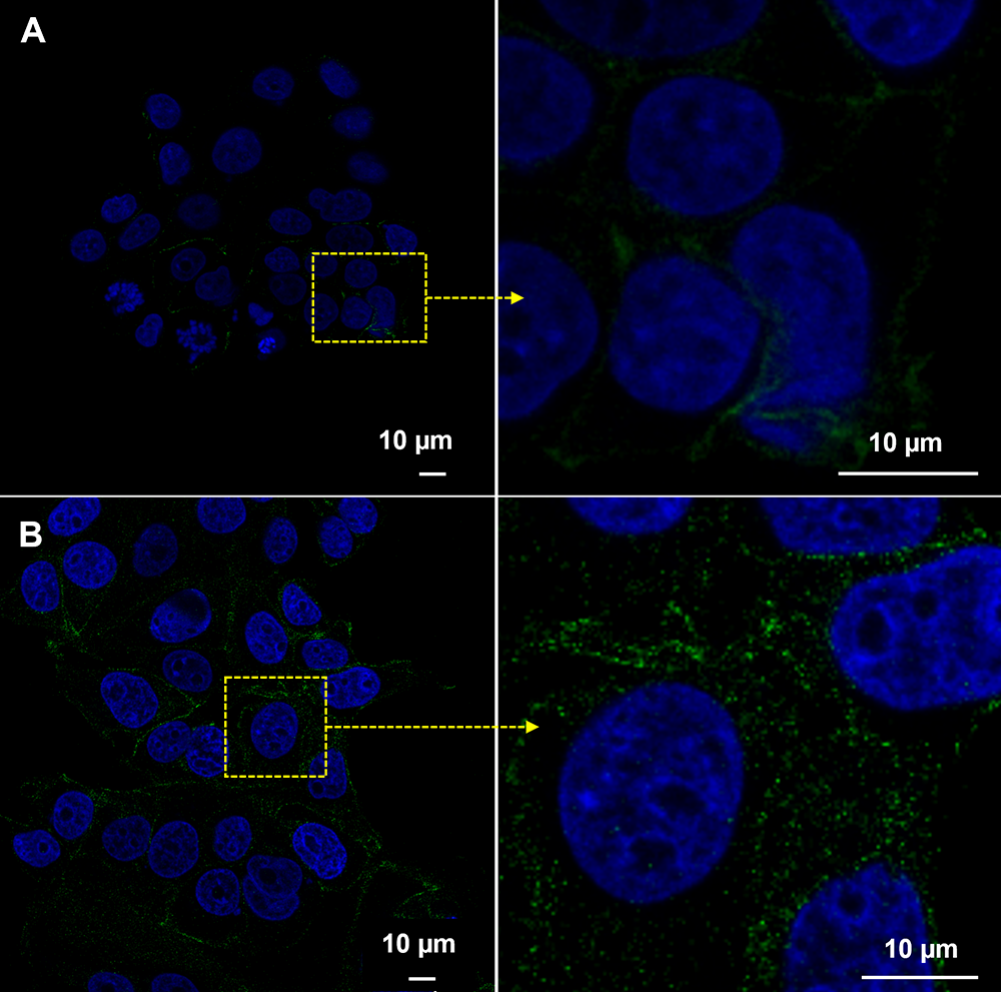
Confocal laser scanning microscopy of the MCF7 cell line after 24 h of exposure to (A) CUR-NCs and (B) FA-NCs. Nuclei were stained with DAPI (blue) and green nanocrystals due to curcumin fluorescence.

Finaly, to obtain a representative and higher-quality image, an imaging study was conducted on the MCF7 cell line using an exposure of 50 μg/mL CUR-NCs and FA-NCs. When cells were exposed to nonfunctionalized NCs (Figure 10A), the green dots representing the NCs were found at a low concentration near the blue-colored nucleus, indicating a restrained entry into the cell. However, in the assay performed with FA-NCs (Figure 10B), the number of green dots inside the cell increased significantly. This further demonstrates the efficacy of the treatment, consistent with the results obtained in the previously mentioned assays.

The NC technology has emerged as a flexible and universal platform for the formulation of poorly soluble actives. However, this work presents a major departure from current NC applications. While NCs administered orally, for instance, may dissolve relatively quickly in large amounts of fluids of the stomach, the literature indicates that NCs may remain as intact particles in the body for variable periods of time, depending on the administration route. Internalization of NCs by cells is an area with a clear knowledge gap. For instance, commercial long-acting intramuscular formulations, such as Invega Sustenna and Cabenuva, form depots in the application site, where infiltrated macrophages engulf drug particles producing a fibrous encapsulation that affects drug release.^68^ Research papers from this group, where drug NCs were loaded into dissolving microneedles, showed drug depots in the rats’ skin for up to 4 weeks. A report from Abbate et al.^28^ described an interesting approach, where microneedle-mediated delivery of positively charged rilpivirine and cabotegravir NCs led to drug accumulation in the central nervous system. Another work from Shen et al.^18^ demonstrated that integral quercetin NCs administered orally were detected in the plasma for up to 48 h. Once internalized by macrophages, NCs are located in the phagolysosome, from where the hydrophobic active eventually dissolves and permeates back to the cytosol and then back to the plasma, modifying the pharmacokinetic performance of the drug.^69^ This phenomenon has been reported to modify bioavailability of intravenously injected itraconazole NCs.^70^ The NCs reported here take advantage of these phenomena, leading to selective accumulation in cancer cells where the drug eventually dissolves and exerts its pharmacological action. This brings to attention another crucial point, which is the biopersistence of NCs. While other nanoparticles made of inorganic materials or nondegradable polymers or lipids might accumulate in the body, or be challenging to remove by glomerular filtration, NCs eventually dissolve, leaving no residues behind, which presents an attractive alternative to conventional nanoparticles.^71^ Interesting work related to the use of etoposide^72^ and fisetin^73^ NCs as potential cancer treatments was found in the literature, with promising outcomes related to an increased dissolution rate of these agents. Again, different from these reports, FA-NCs were used as nanovectors, taking advantage of their high drug payloads.

## 4 Conclusions

The synthesis and characterization of functionalized FA-NCs have been described in this paper for the first time, with the aim of improving the cellular uptake of CUR into cancer cells. To functionalize NCs, a commonly used technique to modify the surface of conventional lipid and polymer nanoparticles, EDC/NHS was successfully applied. The hydrodynamic diameter found for the FA-NCs obtained was 177.8 nm, with a narrow size distribution and zeta potential of 2.7 mV. An enhanced degree of growth inhibition of FA-NCs (compared to that of CUR-NCs) against HeLa and MCF7 cancer cell lines was observed. J774A.1 healthy cell line viability was almost unmodified by treatment with functionalized nanoparticles. The uptake results showed that in both cancer cells, the percentage of entry of FA-NCs was substantially higher than that of naked NCs. In addition, macrophage studies indicated that the entry of FA-NCs was lower than those obtained with the nondecorated NCs, indicating that FA-NCs could circulate for longer periods *in vivo*. These results indicated that the nanovectors developed here have the potential for drug accumulation in cancer tissues, mediated by the overexpressed folate receptors, while evading interception by macrophages and without causing any harm to them. This innovative approach not only sets a pioneering precedent in the NCs field but also holds substantial potential in the field of targeted nanomedicines with application in cancer therapies and a wide range of diseases where selective targeting is required. Further *ex vivo* and *in vivo* experimentation using these active and others will help to validate the technology and facilitate its translation to the clinic.

## Supporting Information Available

Details of the analytical methods used to quantify FA, UV–vis spectral curves of FA and CUR, quantification method linearity, limit of detection (LoD), and limit of quantification (LoQ) (PDF)
