# Supplementary material for: Folic Acid-Decorated Nanocrystals as Highly Loaded Trojan Horses to Target Cancer Cells
Source: Mol Pharm. 2024 Apr 27;21(6):2781–94. doi: 10.1021/acs.molpharmaceut.3c01186 (PMC11151209; doi:10.1021/acs.molpharmaceut.3c01186)
Supplement: Supplementary file 1 — mp3c01186_si_001.pdf [file mp3c01186_si_001.pdf]

Supplementary information

Folic acid-decorated nanocrystals as highly loaded Trojan Horses to target cancer cells

Marta G. Fuster[2], Jiawen Wang[1], Octavio Fandiño[1], Gloria Víllora[2] and Alejandro J. Paredes[1*]

1- School of Pharmacy, Queen's University Belfast, Medical Biology Centre, 97 Lisburn Road, Belfast. BT9 7BL, UK.

2- Department of Chemical Engineering, Faculty of Chemistry, University of Murcia (UMU), Campus de Espinardo, 30100 Murcia, Spain.

***Corresponding author**

Dr Alejandro J. Paredes
Senior Lecturer in Pharmaceutical Sciences
School of Pharmacy
Queen's University Belfast
Medical Biology Centre
97 Lisburn Road
Belfast, BT9 7BL
United Kingdom
Tel: +44(0)2890971061
Email: a.paredes@qub.ac.uk

1. Supplementary results

1.1. Quantification of folic acid by UV-Vis spectrophotometry

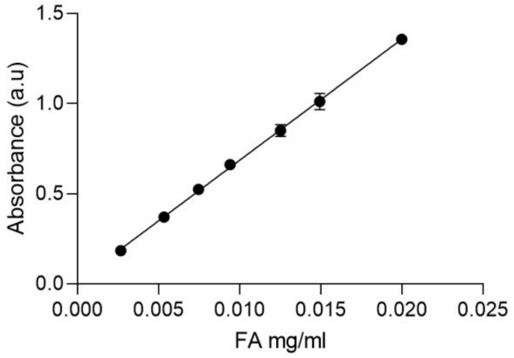

Figure 1. Calibration data set of FA dissolved in DMSO. n=3.

Table 1. Calibration curve parameters.

**Fitting Parameters**	
Slope	67.24 ± 0.67
Y-Intercept	0.01461 ± 0.00787
R^2 *	0.9995
LoD **	4 µg/mL
LoQ ***	1.45 µg/mL

* Linearity, ** Limit of detection, ***Limit of quantification

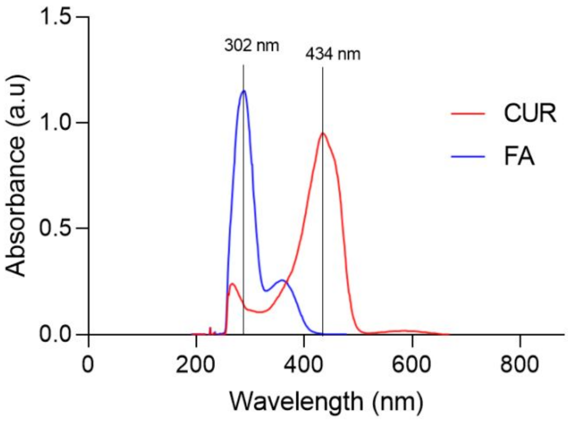

Figure 2. UV-Vis spectral curve of CUR and FA. The wavelength value with the highest intensity is indicated.
